# A Boltzmann model predicts glycan structures from lectin binding

**DOI:** 10.1101/2023.06.03.543532

**Published:** 2023-06-06

**Authors:** A.J. Yom, Austin W.T. Chiang, Nathan E. Lewis

**Affiliations:** 1 Department of Physics, University of California, San Diego; 2 Department of Pediatrics, University of California, San Diego; 3 Department of Bioengineering, University of California, San Diego

## Abstract

Glycans are complex polysaccharides involved in many diseases and biological processes. Unfortunately, current methods for determining glycan composition and structure (glycan sequencing) are laborious and require a high level of expertise. Here, we assess the feasibility of sequencing glycans based on their lectin binding fingerprints. By training a Boltzmann model on lectin binding data, we are able to predict the approximate structures of 90 ± 5% of N-glycans in our test set. We further show that our model generalizes well to the pharmaceutically relevant case of Chinese Hamster Ovary (CHO) cell glycans. We also analyze the motif specificity of a wide array of lectins and identify the most and least predictive lectins and glycan features. These results could help streamline glycoprotein research and be of use to anyone using lectins for glycobiology.

## 1 Introduction

Glycans are diverse polysaccharides found in every branch of life. In humans, they are critical to protein folding, immunity, cell-cell interactions, and many other processes. Improper glycosylation has been implicated in several disease states, and may be useful as an early indicator of cancer [1–10]. Proper glycosylation is vital to the efficacy of many protein therapeutics and vaccines [11–13]. The influence of glycans on protein function and organismal physiology highlights how imperative it is to identify flexible and affordable methods for sequencing glycans.

Currently, the most common method for determining the structures of glycans is to use mass spectrometry (MS) coupled with expert annotation, or potentially automated annotation [14, 15]. Other experimental techniques also exist, such as NMR [16, 17] and exoglycosidase treatment [18, 19]. Unfortunately, all of these methods are expensive, and each requires a high level of expertise to conduct the experiments and interpret the results. Also, with mass spectrometry, it can be difficult to distinguish bond orientations, which can impact physiological responses [20].

By contrast, lectins, antibodies, and other carbohydrate-binding proteins (collectively *lectins* hereafter) often display stereospecific binding, and many mass-produced agents with diverse glycan-binding specificities can be readily obtained [21]. For this reason, they have long been employed to quickly discern the presence or absence of certain glycan motifs. Arrays of lectins have been used to generate binding profiles for glycoproteins, and to assay the corresponding cellular interactions [22–25]. Recent studies have utilized machine learning (ML) and big data techniques to extract the binding specificities of a large number of commonly used lectins [26–29], thereby providing standard references for glycobiologists.

Following the trend of applying ML and big data techniques to glycobiology [30,31], we attack the problem of *de novo* glycan sequencing, i.e., the complete elucidation of an unknown glycan’s composition and structure. Though a single lectin cannot extract nearly as much information as LC-MS, here we test if with a panel of many lectins, and by leveraging the power of machine learning, one could predict a glycan’s full structure from its lectin binding fingerprint.

In this study, we show that a simple Boltzmann machine using a wide panel of lectins can predict the approximate structures of 90 ± 5% of N-glycans from the Consortium for Functional Glycomics (CFG) glycan array. We also demonstrate that our model can predict glycans from Chinese Hamster Ovary (CHO) cells, the primary cell type for producing therapeutic glycoproteins [32]. Then, using information theoretic measures, we identify the most robust lectin-motif binding pairs, which we list as a resource for those interested in experimenting with lectins. We also determine which glycan motifs are most elusive to currently available lectins, thus suggesting profitable directions in lectin engineering or the development of other glycan-binding molecules.

## 2 Results

We first obtained and analyzed lectin binding data from CFG glycan arrays [26] in order to test if a machine learning model can accurately extract and encode the motif-binding patterns of each lectin. We then validated the accuracy of our model on both the CFG glycan set and a comprehensive set of CHO N-linked glycans from two previous studies [26, 33]. The results of these analyses follow.

### 2.1 A Boltzmann model is near-optimal for encoding lectin binding

**Figure 1:**
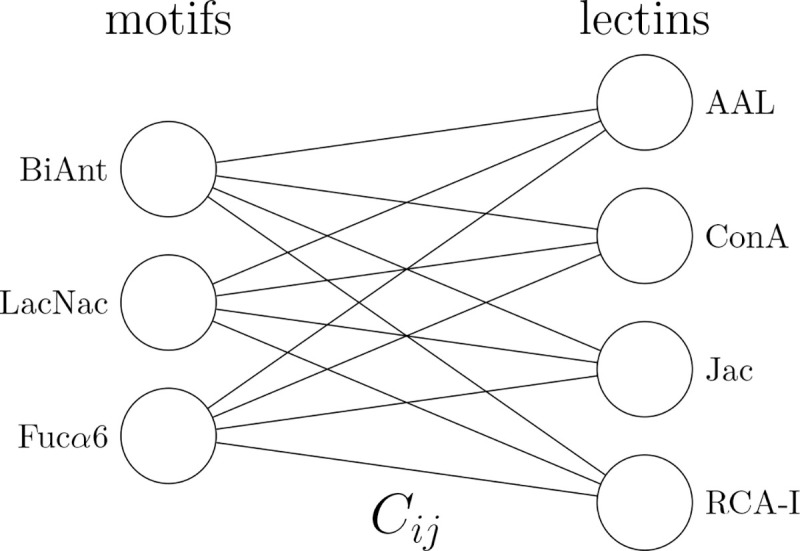
Fully visible Boltzmann machine

In this study, we tested if glycan structures can be predicted from lectin binding patterns. Each lectin has a small set of motifs to which it binds. Since lectins are not expected to have logically complex binding rules, we expect shallow neural network topologies to be adequate. A reasonable minimal model for this interaction would be a fully visible Boltzmann machine, which can be conceptualized as a two layer network (Figure 1). In this model, we generate a likelihood for each possible glycan, based on the lectin binding signal received.

Boltzmann Model:

$$\log P(m|l)=\sum_{i}a_{i}m_{i}+\sum_{ij}m_{i}C_{ij}l_{j}-F$$

where F is a normalization constant.

Could a more complex machine learning architecture, such as a deep neural network, outperform such a simple model? This would be true if, for example, a lectin binds a glycan only when two motifs are present, but not when either one is present. The key property of such a scenario is the presence of higher order interactions, i.e., those involving the product of more than one lectin or more than one motif. One way to parameterize a maximally general model would be to include all higher order interactions. The next level up from our model would be to include only the three-party interactions, i.e. those between two lectins and one motif, and those between two motifs and one lectin.

Second-Order Model:

$$\;\log P(m|l)=\sum_{i}a_{i}m_{i}+\sum_{ij}m_{i}C_{ij}l_{j}-F\;+\sum_{ij}B_{ij}m_{i}m_{j}+\sum_{ijk}D_{ijk}m_{i}m_{j}l_{k}+\sum_{ijk}E_{ijk}m_{i}l_{j}l_{k}$$

where F is a normalization constant.

**Figure 2:**
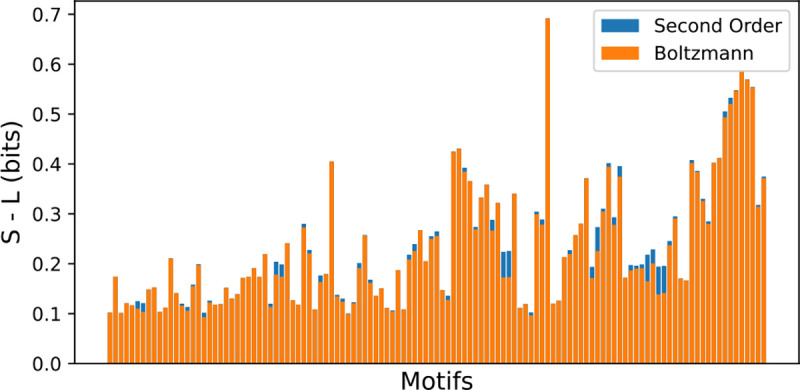
Comparison of extractable information about each motif in the Boltzmann and Second-Order Models. Evidently, with regard to the task of predicting any single motif, there is little to be gained from using the higher order model. See Methods 5.7 for more details.

One way to gauge the utility of adding these extra terms would be to measure the impact of these terms on the amount of information extracted about any one motif. In general, we find that our minimal model is virtually indistinguishable from the Second-Order model in this sense (Figure 2). Therefore, if we assume similar diminishing gains from adding further higher order terms, we can reasonably conclude that our simple Boltzmann model is near-optimal at encoding lectin binding patterns.

### 2.2 The model predicts glycan structure from lectin profiles

Given binding measurements from a panel of lectins, our model will predict the structures of glycans being bound. Since our model is probabilistic, each glycan will be predicted with an associated likelihood. These likelihoods can then be used to rank the glycans, with rank 1 glycans being the model’s best guess, rank 2 being the second most likely glycan, and so on. We are interested not only in how often our model predicts the exact correct glycan, but also in how often the correct glycan is among its top n predictions.

We begin by generating a set of glycans sufficiently large and diverse as to encompass all CFG glycans and all other glycans that could plausibly exist given the linkage patterns found in the CFG dataset. As we describe in Methods 5.4, this can be achieved by randomly sampling a minimal Markov model. We find that we must only generate on the order of 10^4^ glycans to cover the full set of plausible glycans. For each lectin profile, a likelihood is calculated for all generated glycans, in addition to all CFG glycans. Since the predicted structures can come from a diverse set of glycans not found in the CFG array, the prediction can be considered an act of *de novo* glycan sequencing.

**Figure 3:**
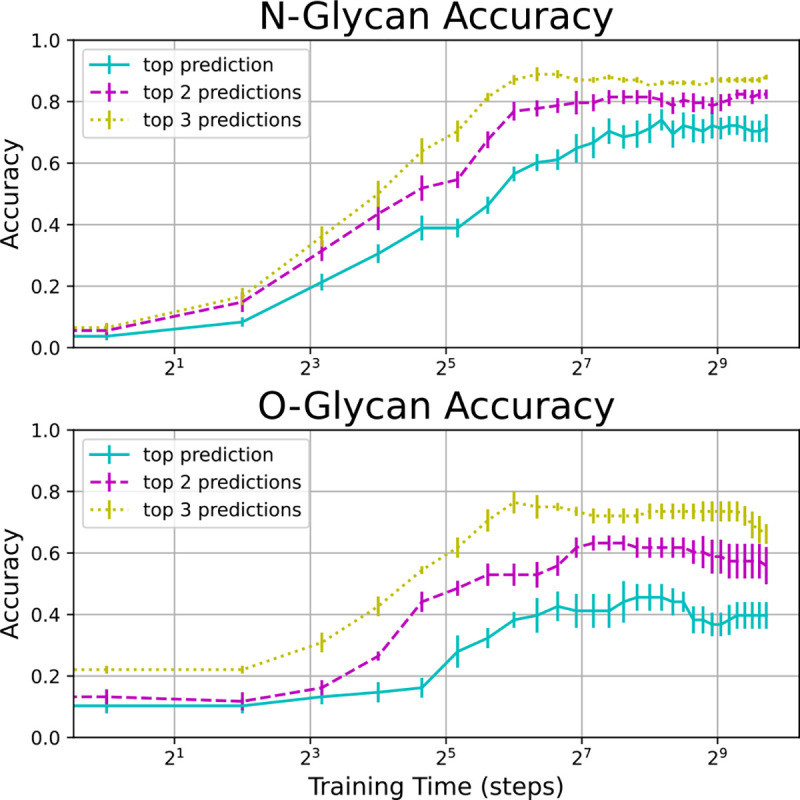
Test accuracy on N-linked CFG glycans. Results averaged over 16 training/test set partitions. Error bars represent the standard error.

To test the model’s glycan prediction accuracy, we separated the Consortium for Functional Glycomics (CFG) glycans into training and test sets, and then trained and tested our model on the corresponding binding data from 73 lectins and antibodies [26]. Our model was highly predictive on N-glycans, predicting the exact structure for 66 ± 5% of glycans, and including the correct structure in its top three predictions for 90 ± 5% of glycans (Figure 3). Our model was less predictive on O-glycans, but still predicted the exact structure of about half of the glycans tested. We speculate that this may be due to variable presentation on the array. Since the N-core is large, lectins bind N-glycans further from the anchor point, and could therefore perhaps be more insulated from such effects. In either case, even when the model fails to identify the correct glycan, the outputted predictions are very similar to the correct glycan, particularly in terms of their terminal motifs (Figure S-4).

### 2.3 Model generalizes to CHO cell glycans

The primary value of a machine learning model is its ability to generalize to new contexts. We have thus far only demonstrated our model’s accuracy on CFG array glycans. These arrays house a diverse set of glycans that could appear in many different organisms and contexts.

By contrast, glycans restricted to a single species or cell type will display many constraints on their structure [34–36], and may carry motifs underrepresented in the CFG array, which can affect our model’s accuracy. Accordingly, we sought to test our model on glycans from both recombinant and naturally occurring glycoproteins produced in CHO cells.

From a pharmaceutical point of view, this question matters because CHO cells are the most commonly used cells in protein drug production lines [32]. A carefully curated list has been reported, which contains all N-linked glycans that have thus far been reported on proteins produced by CHO cells in meaningful quantities [33]. Will a Boltzmann model trained on CFG glycans be able to sequence these CHO cell glycans?

To test this, we used the CFG glycan arrays as the training set, but excluded glycans that appear in the CHOGlycoNET dataset. Our test set includes all glycans that appear in the CHOGlycoNET dataset and for which we have lectin binding data. Only 21 glycans were found both on the CFG glycan arrays and CHOGlycoNET, roughly 5% of the CFG dataset or 12% of the CHOGlycoNET dataset. One may therefore reasonably expect this underrepresented subset of glycans to be difficult to generalize to.

**Figure 5:**
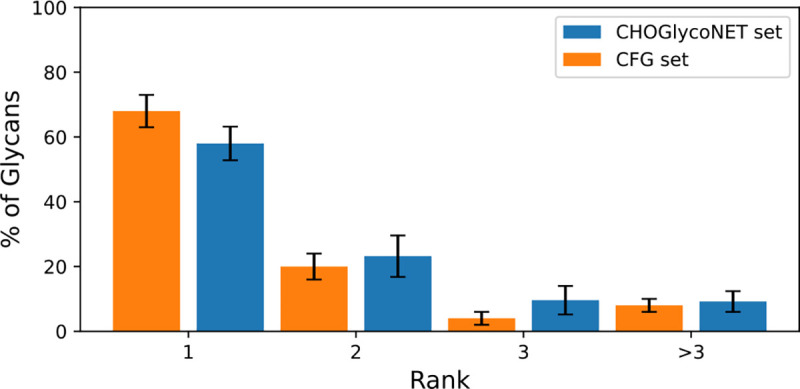
Ranks returned for each correct CHO N-glycan alongside the accuracies for general CFG N-glycans. Note that over 90% of glycans are found in the top 3 predictions. Averages taken over 16 runs.

Despite this small overlap, our model was remarkably predictive, exactly identifying 14 / 25 of glycans, and placing 23 / 25 glycans in the top 3 predictions (Figure 5). This could be due to the reduced complexity of N-glycosylation, particularly when confined to a single cell type. If the model were trained on lectin data taken from a large CHO glycan set, we would expect to witness even higher accuracy.

### 2.4 Some motifs are invisible to lectin binding

Not all common glycan motifs can be identified with the set of lectins we studied here. In general, terminal motifs were the best identified, presumably because they are least susceptible to steric interference. This is in broad agreement with most known lectin binding rules [26, 28, 37, 38].

**Figure 6:**
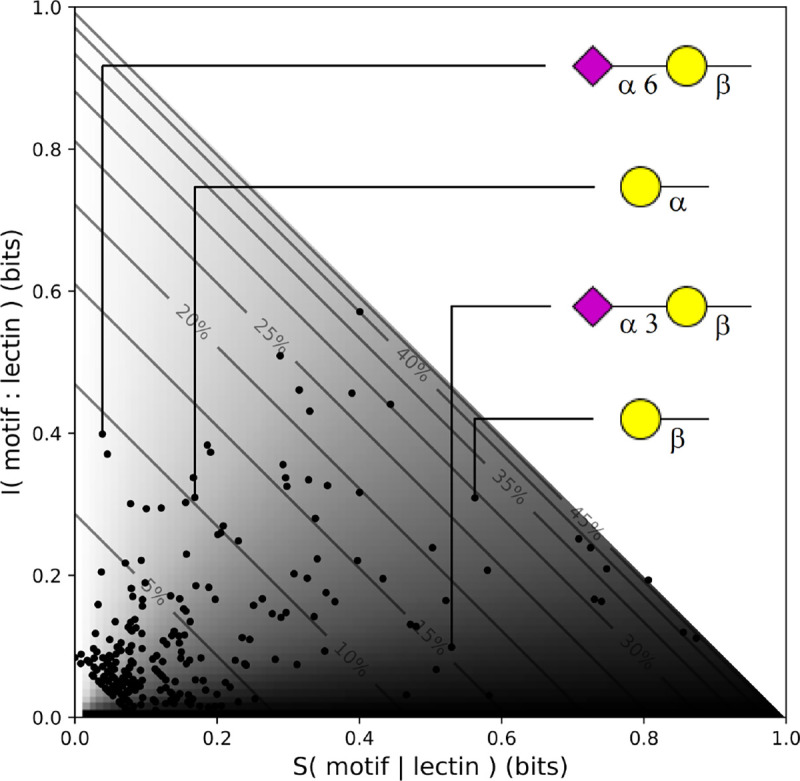
Scatter plot of mutual information and conditional entropy for each motif and its best binding lectin. Motifs on the light side are well-recognized, while motifs on the dark side are invisible to our lectin array. Contours correspond to the prevalence of each motif in our dataset, with the least common motifs occurring in the bottom left corner. Note that variations in bond orientation can have dramatic impacts on motif recognition.

We observed, however, that there are also terminal motifs that are less well-identified here, and finding lectins that recognize these features could improve performance of our model in future studies. The following is a list of motifs that we believe may be identifiable with different lectins:

- Terminal Neuα3Gal, for which many binding lectins have already been reported but were not included in our dataset [38–43]. Meanwhile, the similar Neuα6Gal, is one of the most sharply identified motifs in our dataset (Figure 6), demonstrating one of the key advantages of lectin analysis: *bond specificity*.
- Terminal Fucα2Galβ, which is recognized in some contexts (i.e. BgH) but not in others.
- Terminal Galβ, which has reportedly been recognized by BPL [26], but which we found only to be the case in N-glycans.
- Internal polyLacNAc, which is recognized by several lectins, but not reliably.

As noted, some of these motifs can already be recognized by commercially available lectins not found in our data. Others could potentially be identified using engineered glycan-binding molecules such as nanobodies [44–47]. By progressively illuminating these dark corners of the motif space we can further enhance the information content of our array.

### 2.5 Robust motif binding is rare among lectins

The binding motifs of many lectins have been studied and catalogued [26–29, 38], and experiments have been performed to evaluate the relative affinities of lectins for specific glycoproteins of interest [48–50]. However to date, little has been published to quantify the specificity of lectin binding and only ever for a predetermined set of known glycan motifs [37, 51]. In this study we extract all possible statistically significant motifs (see Methods 5.2), and quantify the specificity of lectin binding for each of these motifs using the mutual information, which has several desirable properties (see Methods 5.6).

**Figure 7:**
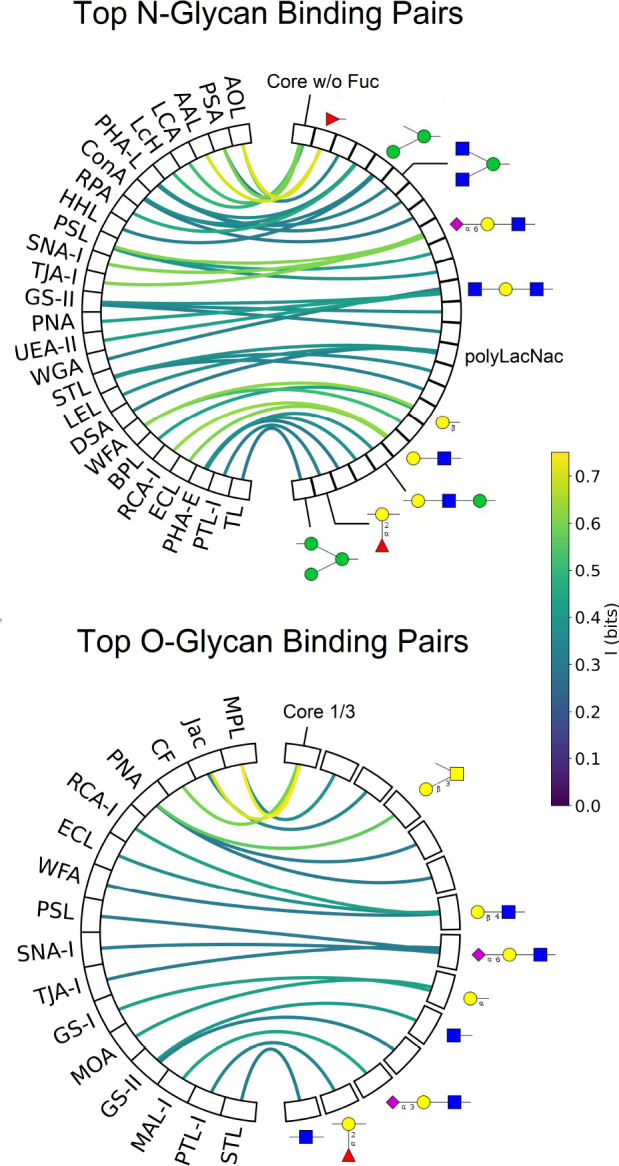
Mutual information between lectins and motifs. Only pairs with ≥ .3 bits of information are displayed. To save space, for each lectin, only the highest correlation motif is displayed.

In our analysis, we find a wide range of lectin accuracies, with only a handful of lectins binding any motif robustly (Figure S-1, Figure S-5). This corroborates earlier findings [51]. Figure 7 shows the best lectins for each significant motif in the cases of N and O glycosylation.

### 2.6 Most lectins only reliably bind one motif

**Figure 8:**
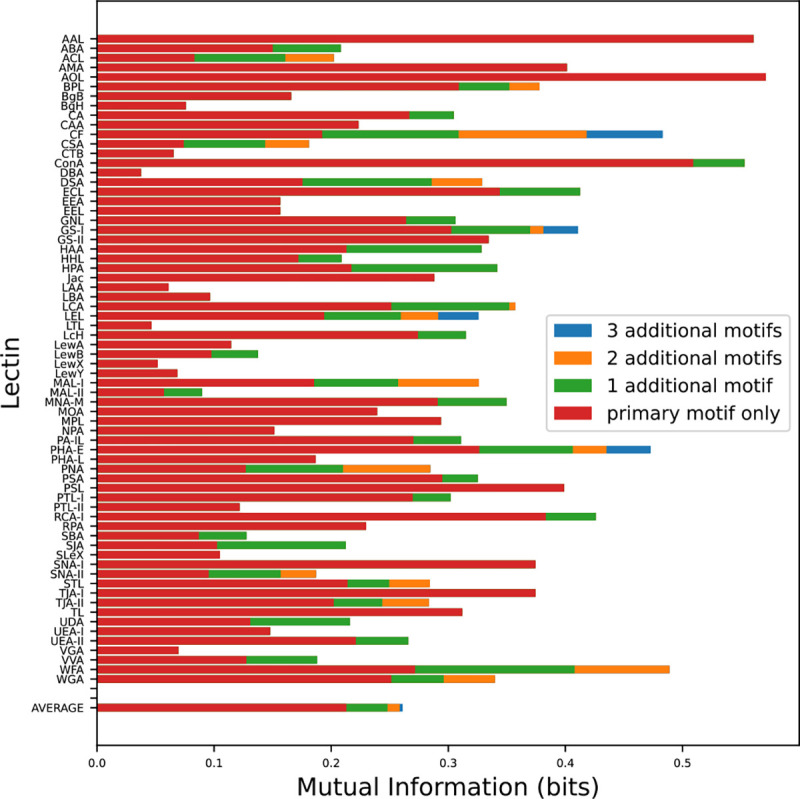
Mutual informata between lectins and their best-binding motifs. On average, 82 % of a lectin’s captured information comes from its primary binding motif. This increases to 86% and 89% when restricting to N and O glycans respectively, due to increased specificity.

Efforts have recently been made to identify the secondary and tertiary binding motifs of many lectins, i.e. those additional motifs which lectins may recognize with less affinity or frequency than their primary binding moieties [26, 37]. To test if these secondary motifs are predictive of lectin binding, we measured the increase in mutual information gained by adding additional motifs. On average, we found that over 80% of the information captured by a lectin comes purely from its primary binding motif, with additional motifs contributing little extra information (Figure 8).

This fact has positive and negative implications with regard to using lectins to determine glycan structure. On the one hand, it is unlikely that a single lectin can be used to gather information on the presence of multiple motifs. On the other hand, it makes endeavors to interpret lectin binding results much simpler, particularly for a machine learning algorithm.

Our results are consistent with the conclusion that any secondary binding motifs that exist are too closely correlated with their primary binding motifs to generate much extra predictive power. It is also possible that there are secondary motifs that do not appear in sufficient quantity in our dataset to be statistically significant, but would become relevant in datasets where they are more common. In either case, for most lectins, primary binding will capture the lion’s share of the available information.

## 3 Discussion

In this study we analyzed the information content of lectin binding, and demonstrated its utility for de novo glycan sequencing. We have shown that sequencing of CHO N-glycans is possible with current lectin panels, and that a broader range of glycans can likely be sequenced if care is taken to curate a diverse set of motif-specific lectins. We have also shed light on which lectins are most dependable, and which motifs are least identifiable, both of which could be of use to future researchers interested in using lectins for their studies. Our analysis is very general and can be used as a reference for choosing the best set of lectins for any task.

A straightforward way to improve upon our results would be to utilize a more comprehensive lectin panel. We hope that by identifying the motifs least recognized by our current lectin set we have provided a roadmap for future researchers to improve upon our results. As we mentioned earlier, for some of the dark motifs we identified, lectins already exist that can bind them. In other cases, our analysis could guide the engineering of new glycan-binding proteins.

With modern synthetic glycobiology techniques, it is now possible to engineer proteins which bind specific glycans [45–47, 52, 53]. Aptamers, lamprey antibodies, and glycan-binding nanobodies are just a few classes of molecules that can be employed to assay glycan motifs today [44, 54–57]. We hope that our identification of dark motifs can shine a spotlight on fruitful future endeavors in this field.

Since our results were obtained by measuring lectin binding on glycan arrays, our findings should apply broadly to the gamut of lectin array techniques available today. Microfluidic integration and other refinements have made these microarrays high-throughput, reproducible, and tailorable to many lectin sets [58–62]. An intriguing alternative approach put forth recently by Oinam and Tateno [63] would be to use multiplexed DNA-barcoded lectins in solution, and perform next generation sequencing to read out bindings. This would have the benefit of presenting lectins in solution, which avoids the problems of array presentation and allows for flexible use cases.

A critical next question to answer is whether this technology can be used to resolve mixtures of glycans into their component parts. Though at first blush this problem may seem highly underconstrained, there are many biological constraints on glycan construction which narrow the set of plausible glycoprofiles [34–36]. Often, there will only be a few glycans present in appreciable quantities in a sample, differing only by the final monosaccharides in a chain. It is therefore conceivable that lectins in an biological context could be sufficiently accurate for this task.

Despite its ubiquity in nature, glycobiology has long remained insulated from the broader scientific community. It is our hope that by making glycoprofiling more efficient and accessible to non-experts, we can better share what our field has to offer and reap the rewards of integrative science. Glycosylation should not be seen as an excess complication, it should be seen as an opportunity for discovery.

## 5 Methods

### 5.1 Data processing

For this analysis, we utilized published lectin binding data from CFG arrays [26]. Glycans from the CFG Mammalian Glycan Array were filtered to contain only fucose (Fuc), galactose (Gal), N-acetylgalactosamine (GalNAc), glucose (Glc), N-acetylglycosamine (GlcNAc), mannose (Man), and sialic acid (Neu) residues, since these were the only monosaccharides in sufficient abundance in the CFG array for meaningful statistics to be extracted.

Additionally, we discarded glycan fragments fewer than 3 monosaccharides long since they did not possess sufficient complexity, and we ignored modifications such as sulfurylation and phosphorylation. Although these modifications are known to impact lectin binding [37, 47], they were not common enough in our data for a statistical analysis. Incorporation of these features into future studies should result in greater glycan resolution.

### 5.2 Motif recognition

Motifs were taken to be any connected subgraph of any glycan which appeared more than 5 times in our dataset. Motifs of size > 5 monosaccharides were removed to reduce computational load. Inclusion or exclusion of these large motifs was not found to impact performance.

### 5.3 Binarization of lectin binding

Since lectin binding strength can vary wildly depending on context for a given motif, linear binding relationships are not appropriate (Figure S-1). Instead, we binarize our lectin binding data by generating a range of thresholds from 1000 to 20,000 RFU to serve as binding cutoffs. If the raw binding value in RFU obtained from the array for any lectin-glycan pair exceeds the threshold, we record this as a discrete binding event. Therefore, for each lectin, we will have several binarized binding values corresponding to each threshold and lectin concentration.

### 5.4 Markov Modelling of Glycan Space

Markov models of varying complexity have been used often in glycobiology [36,64,65] Here we use a simple model that takes the previous two residues in a chain and predicts the next one. This is less sharp than other models which have been used because the goal is not to exactly model the glycan space, which may be constrained in many ways, but to provide a very broad range of glycans which could possibly exist. This will allow us to measure the de novo sequencing accuracy, and later constrain the space further in a cell or species specific manner to obtain greater accuracy.

We found our model to produce reasonable and diverse results (Figure S-2, Figure S-3), and calculated its entropy to be 12.3 bits based on the CFG glycan set. This implies that a random sample of on the order of 10^4^ glycans should provide good coverage of the full range of possible CFG-like glycans. An even more general model would take only a single monosaccharide as input, but we found this to produce pathological results (Figure S-2).

### 5.5 Model training

Since our Boltzmann model is convex, all training procedures should converge to the same unique maximum likelihood solution. We used gradient descent with step size .1. We did not find L1 or L2 regularization terms to make a meaningful difference in our results, so we did not utilize them in any of the figures presented here.

### 5.6 Mutual Information

One way to measure the sharpness of a lectin at recognizing a motif would be to use the correlation coefficient; however this suffers from three drawbacks: First, a linear measure is not appropriate because the binding strength can vary wildly for the same motif in different contexts. Second, a high correlation coefficient can be achieved when a rarely binding lectin overlaps by chance with a rarely expressed motif. It would then require further parameters (p or σ for example) to determine whether this binding is actually statistically significant. Third, in a scenario we examine in our paper, if a lectin binds multiple motifs it may not be well correlated to any one of them.

One measure that overcomes these deficiencies is the mutual information:

I(A:B)=S(A)+S(B)−S(AB)

This measure has many desirable properties: First, it is measured in bits, enabling us to compare the amount of information obtained to the amount of information left unknown. Second, S(A)≥I(A:B)≤S(B). This means that rare, statistically insignificant motifs yield insignificant mutual informata. Third, A and B need not be a single lectin or motif. This will allow us to assess the impact of binding patterns involving more than one lectin or motif.

### 5.7 Extractable information

Any probability model’s accuracy can be assessed using the Kullback-Leibler Divergence (KLD). If we restrict our attention to the problem of predicting the presence or absence of a single glycan motif, then our classification is binary and the KLD reduces to the logistic loss function:

$$\left.L(p)=-\frac{1}{N}\left(\sum_{i\in G}\;logp_{i}+\sum_{i\notin G}\;log\left(1-p_{i}\right)\right)\right)$$

where $p_{i}$ denotes the model’s predicted probability that glycan i possesses the desired motif, G denotes the set of glycans with said motif, and N denotes the total number of glycans.

We consider the case of using any two lectins to predict a given motif. We may then define zeroth, first, and second order models as follows:

$$logp^{0}=a^{0}$$

$$logp^{1}=a^{1}+b^{1}l_{1}+c^{1}l_{2}$$

$$logp^{2}=a^{2}+b^{2}l_{1}+c^{2}l_{2}+d^{2}l_{1}l_{2}$$

where $l_{1}$ and $l_{2}$ denote the lectin binding binaries and we optimize the parameters a,b,c,d to minimize the logistic loss.

The difference S−L may be used as a measure of the information extracted. The zeroth order model takes no lectin inputs, so $S-L\left(p^{0}\right)=0$. The second order model is fully general for two lectins, so $S-L\left(p^{2}\right)=I\left(m\;:l_{1},l_{2}\right)$. The first order model extracts an amount of information in between these two limits, and as we show in Figure 2, comes quite close to the second order model.

## Supplementary Material

Supplement 1

## Figures and Tables

**Figure 4:**
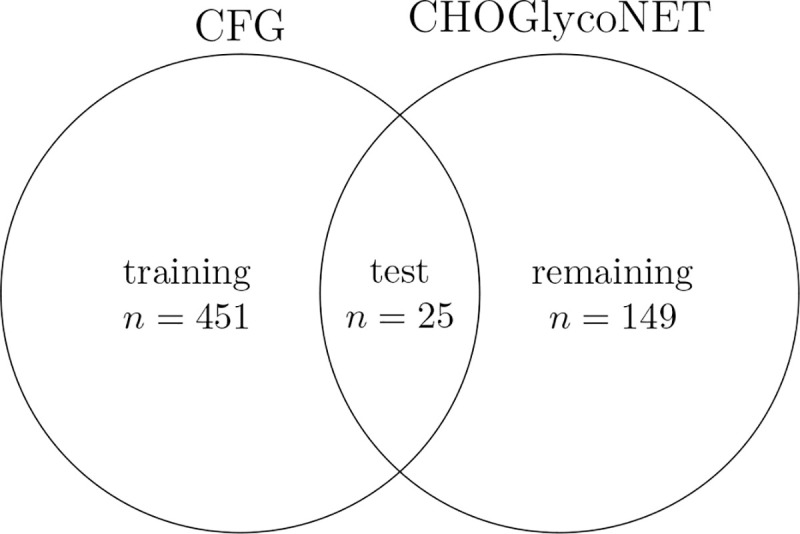
Training and test partitions of CFG and CHOGlycoNET glycans. n denotes the number of glycans in each set.
