# Supplementary material for: A Boltzmann model predicts glycan structures from lectin binding
Source: bioRxiv. 2023 Jun 6:2023.06.03.543532. Preprint. [Version 1] doi: 10.1101/2023.06.03.543532 (PMC10274649; doi:10.1101/2023.06.03.543532)
Supplement: Supplementary file 1 [file NIHPP2023.06.03.543532v1-supplement-1.pdf]

bioRxiv preprint doi: https://doi.org/10.1101/2023.06.03.543532; this version posted June 6, 2023. The copyright holder for this preprint (which was not certified by peer review) is the author/funder, who has granted bioRxiv a license to display the preprint in perpetuity. It is made available under aCC-BY 4.0 International license.

6 Supplemental Materials

6.1 Lectin Code Glossary

Code	Full Name	Code	Full Name
AAA	Anguilla Anguilla Agglutinin	MAL-II	Maackia Amurensis II
AAL	Aleuria Aurantia Lectin	MNA-M	Morniga-M
ABA	Agaricus Bisporus Agglutinin	MOA	Marasmius Oreades
ACL	Amaranthus Caudatus	MPL	Maclura Pomifera
AMA	Arum Maculatum	NPA	Narcissus Pseudonarcissus
AOL	Aspergillus Oryzae Lectin	PA-IL	Pseudomonas Aeruginosa IL
BPL	Bauhinia Purpurea	PHA-E	Phaseolus Vulgaris E
BgB	Blood Group B Antibody	PHA-L	Phaseolus Vulgaris L
BgH	Blood Group H Antibody	PNA	Peanut Agglutinin
CA	Colchicum Autumnale	PSA	Pisum Sativum Agglutinin
CAA	Caragana Arborescens	PSL	Polyporus Squamosus
CF	Codium Fragile	PTL-I	Psophocarpus Tetragonolobus I
CSA	Cytisus Scoparius	PTL-II	Psophocarpus Tetragonolobus II
CTB	Cholera Toxin B	WA	Phytolacca Americana
ConA	Concanavalin A	RCA-I	Ricinus Communis
DBA	Dolichos Biflorus	RPA	Robinia Pseudoacacia
DSA	Datura Stramonium	SBA	Soybean Agglutinin
ECL	Erythrina Cristagalli	SJA	Sophora Japonica
Fors	Forssman Antibody	SLeA	Sialyl Lewis A Antibody
GNL	Galanthus Nivalis	SLeB	Sialyl Lewis B Antibody
GS-I	Grifonia Simplicifolia I	SLeX	Sialyl Lewis X Antibody
GS-II	Grifonia Simplicifolia II	SLeY	Sialyl Lewis Y Antibody
HAA	Helix Aspersa Agglutinin	SNA-I	Sambucus Nigra I
HHL	Hippeastrum Hybrid	SNA-II	Sambucus Nigra II
HPA	Helix Pomatia Agglutinin	STL	Solanum Tuberosum
Jac	Artocarpus Integrifolia (Jacalin)	TJA-I	Trichosanthes Japonica Agglutinin I
LAA	Laburnum Alpinum	TJA-II	Trichosanthes Japonica Agglutinin II
LCA	Lens Culinaris Hemagglutinin	TL	Tulipa Lectin
LEL	Lycopersicon Esculentum	UDA	Urtica Dioica
LTL	Lotus Tetragonolobus	UEA-I	Ulex Europaeus Agglutinin I
LcH	Lens Culinaris Hemagglutinin	UEA-II	Ulex Europaeus Agglutinin II
LewA	Lewis A Antibody	VGA	Vicia Graminea Lectin
LewB	Lewis B Antibody	VVA	Vicia Villosa
LewX	Lewis X Antibody	WFA	Wisteria Gloribunda
LewY	Lewis Y Antibody	WGA	Wheat Germ Agglutinin
MAL-I	Maackia Amurensis I		

bioRxiv preprint doi: https://doi.org/10.1101/2023.06.03.543532; this version posted June 6, 2023. The copyright holder for this preprint (which was not certified by peer review) is the author/funder, who has granted bioRxiv a license to display the preprint in perpetuity. It is made available under aCC-BY 4.0 International license.

6.2 Figures

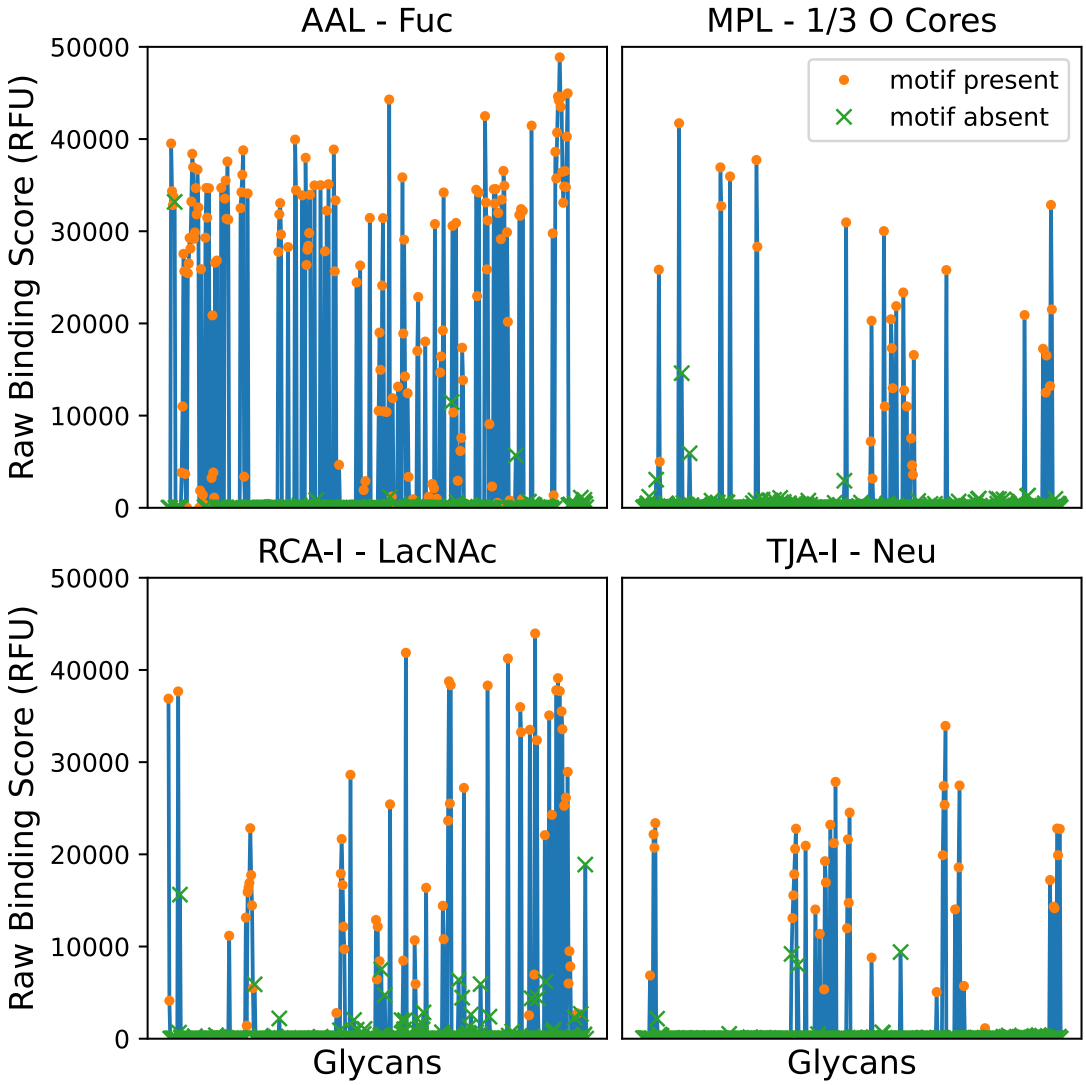

Figure S-1: Raw binding values from our CFG arrays for four of our sharpest lectin-motif pairs. AAL binds Fucα, MPA binds O glycan cores 1 and 3. RCA-I binds terminal Galβ4GlcNac, and TJA-I binds Neuα6Galβ4GlcNAc. Note that even among cases when the motif is present, binding strength can vary by a factor of 2 of more. Also note that the apparent lack of variance in AAL binding strength is due to saturation.

bioRxiv preprint doi: https://doi.org/10.1101/2023.06.03.543532; this version posted June 6, 2023. The copyright holder for this preprint (which was not certified by peer review) is the author/funder, who has granted bioRxiv a license to display the preprint in perpetuity. It is made available under aCC-BY 4.0 International license.

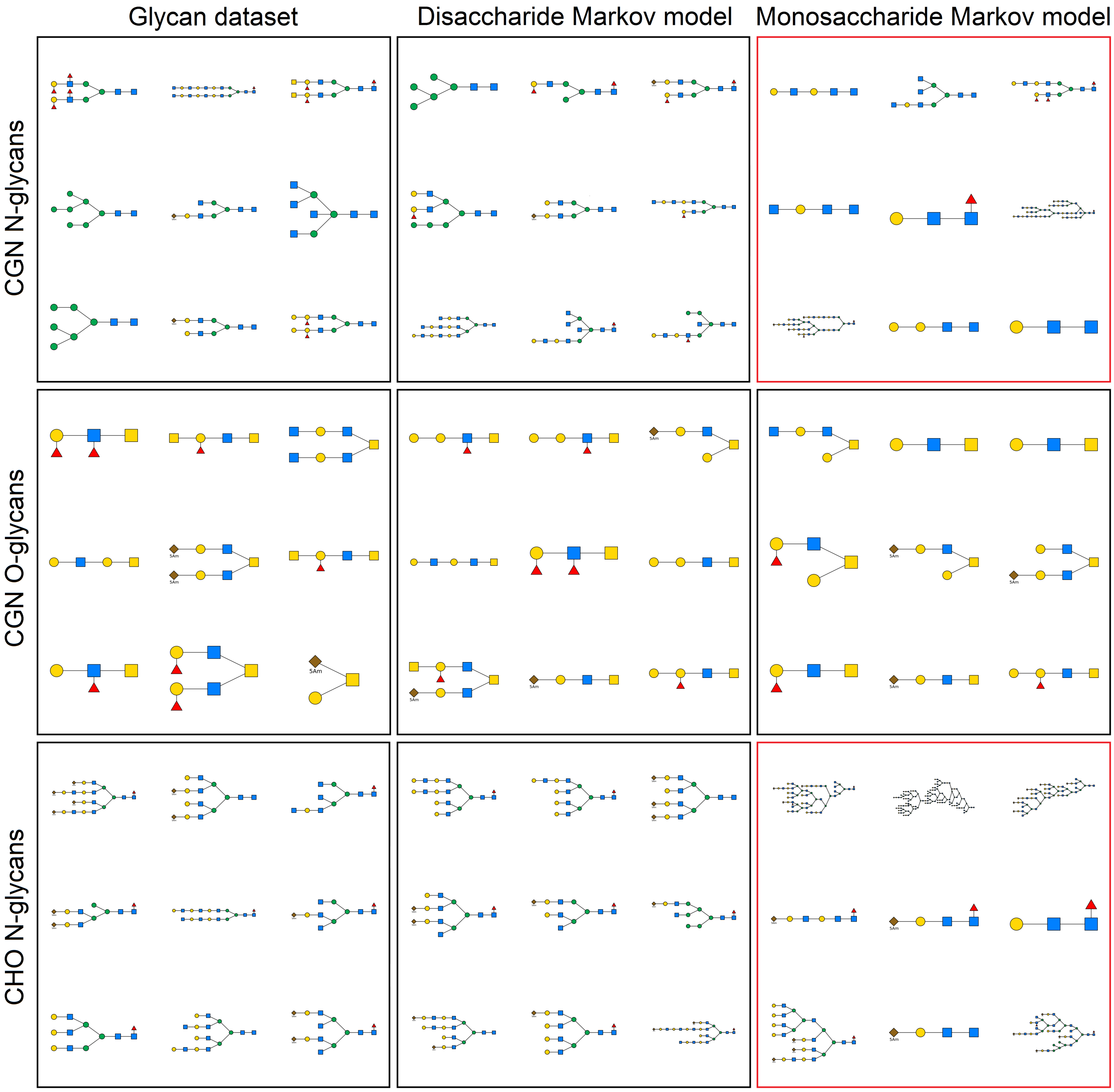

Figure S-2: Random sample glycans from each of our datasets alongside random samples from their respective Markov models. Note that while the disaccharide model used in our paper appears to model the glycans well and produce diverse results, the more general monosaccharide model produces pathological samples on both N-glycan sets.

bioRxiv preprint doi: https://doi.org/10.1101/2023.06.03.543532; this version posted June 6, 2023. The copyright holder for this preprint (which was not certified by peer review) is the author/funder, who has granted bioRxiv a license to display the preprint in perpetuity. It is made available under aCC-BY 4.0 International license.

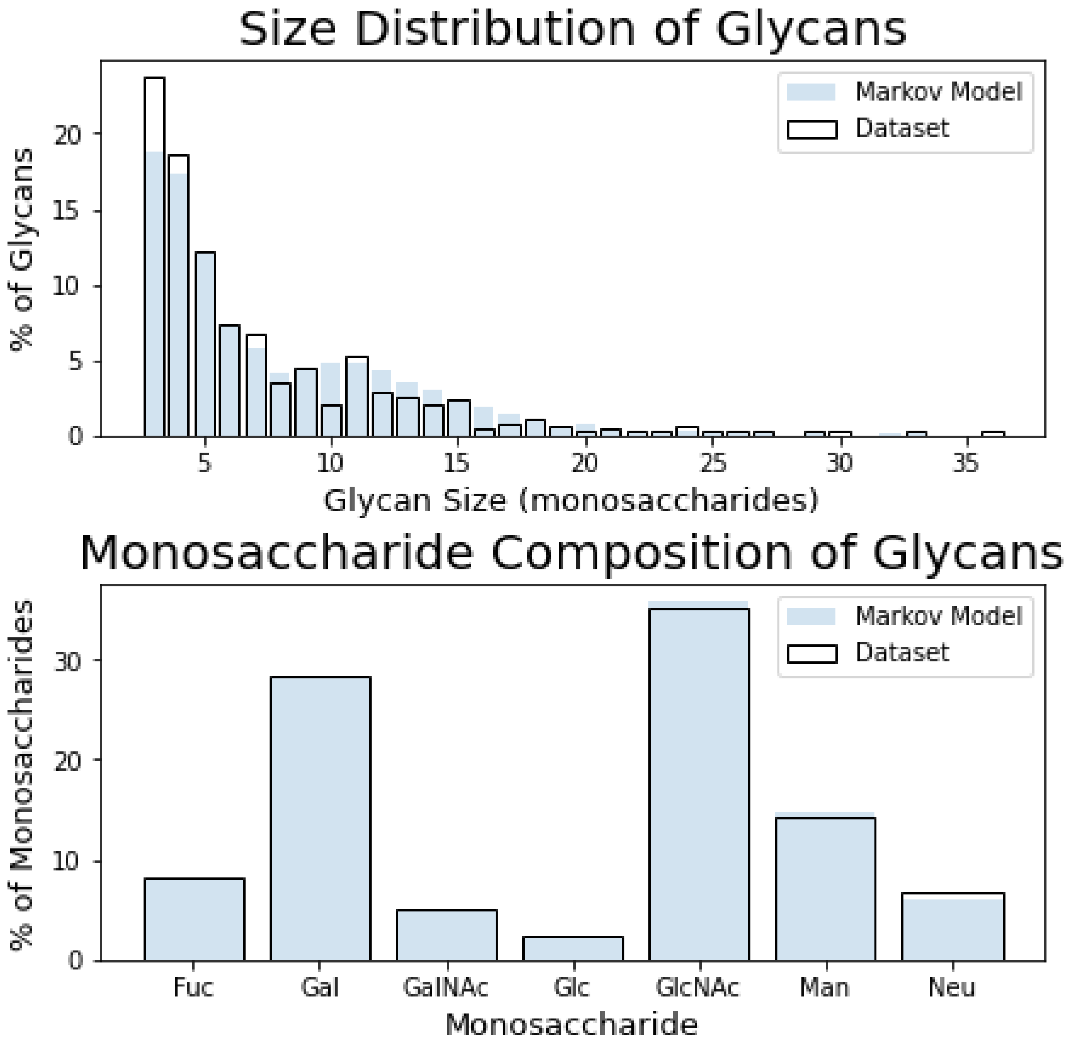

Figure S-3: 10,000 samples from Markov model of CFG glycan set. Our model appears to generate glycans of the right size and sugar composition.

bioRxiv preprint doi: https://doi.org/10.1101/2023.06.03.543532; this version posted June 6, 2023. The copyright holder for this preprint (which was not certified by peer review) is the author/funder, who has granted bioRxiv a license to display the preprint in perpetuity. It is made available under aCC-BY 4.0 International license.

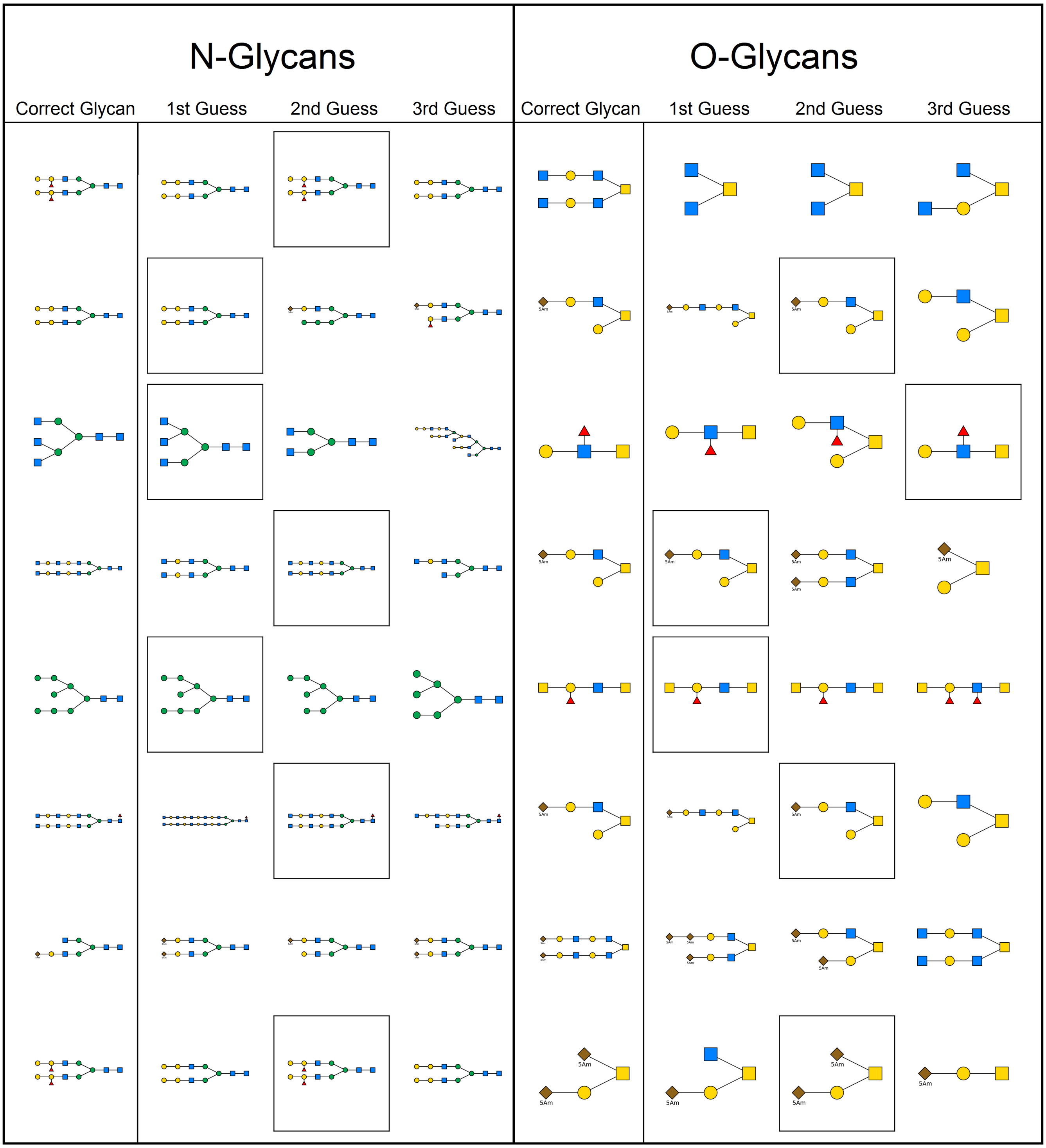

Figure S-4: Random sample of N and O glycans, along with our model's predictions based on their respective lectin profiles. The correct glycan is boxed. Note that even when the correct glycan is not the model's top pick, it is often in the top three predictions. Also note that the predicted glycans are typically very similar to the correct glycan, particularly in terms of their terminal motifs.

bioRxiv preprint doi: https://doi.org/10.1101/2023.06.03.543532; this version posted June 6, 2023. The copyright holder for this preprint (which was not certified by peer review) is the author/funder, who has granted bioRxiv a license to display the preprint in perpetuity. It is made available under aCC-BY 4.0 International license.

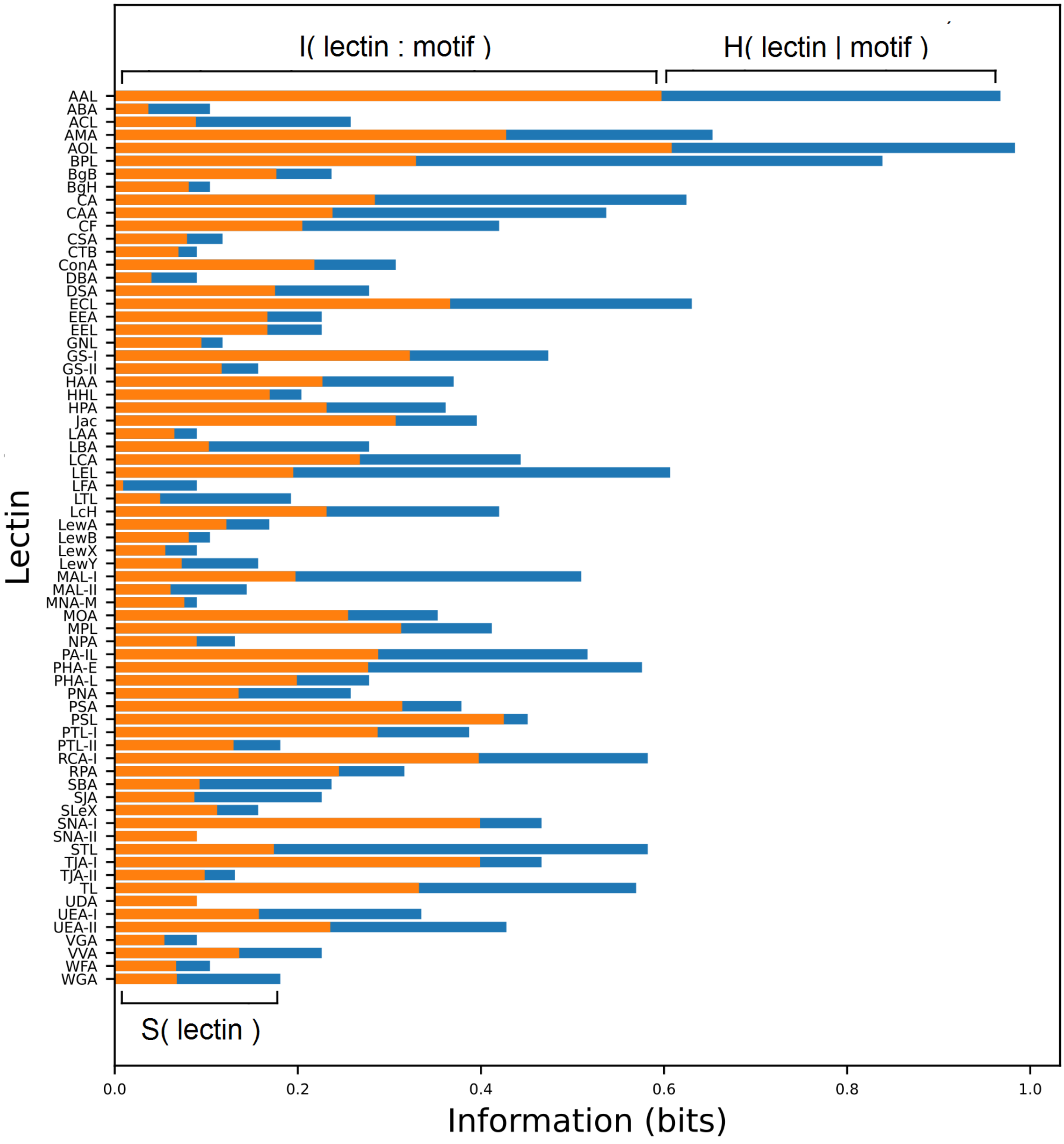

Figure S-5: Information extracted by each lectin about its most sharply bound motif. When the conditional entropy is small compared to the mutual information, the binding is very specific. Note that in most cases, either the mutual information is low, implying that the relevant motif is uncommon, or the conditional entropy is high, implying that the binding is not very robust.
